# High-Purity Fucoxanthin Can Be Efficiently Prepared from *Isochrysis zhangjiangensis* by Ethanol-Based Green Method Coupled with Octadecylsilyl (ODS) Column Chromatography

**DOI:** 10.3390/md20080510

**Published:** 2022-08-11

**Authors:** Gengjie Zhuang, Yuemei Ye, Junling Zhao, Chengxu Zhou, Junwang Zhu, Yanrong Li, Jinrong Zhang, Xiaojun Yan

**Affiliations:** 1 College of Food and Pharmaceutical Sciences, Ningbo University, Ningbo 315211, China; 2 Ningbo Institute of Oceanography, Ningbo 315832, China; 3 Key Laboratory of Applied Marine Biotechnology of Ministry of Education, Ningbo University, Ningbo 315211, China

**Keywords:** microalgal culture, ethanol extraction, ODS column chromatography, ethanol precipitation, *Isochrysis zhangjiangensis*

## Abstract

The exploitation of new economically valuable microalgae as a sustainable source of minor high-value products can effectively promote the full utilization of microalgae. The efficient preparation of minor products from microalgae remains the challenge, owing to the coexistence of various components with a similar polarity in the microalgae biomass. In this study, a novel approach based on the sustainable-oriented strategy for fucoxanthin (FX) production was proposed, which consisted of four steps, including the culture of microalga, ethanol extraction, ODS column chromatography, and ethanol precipitation. The high-purity FX (around 95%) was efficiently obtained in a total recovery efficiency of 84.28 ± 2.56%. This study reveals that *I. zhangjiangensis* is a potentially promising feedstock for FX production and firstly provides a potentially eco-friendly method for the scale-up preparation of FX from the microalga *I. zhangjiangensis*.

## 1. Introduction

Microalgae are considered as one of the most promising substitutes for the comprehensive utilization of agricultural and industrial wastewater and the sustainable production of high-value commercial products [1]. This occurs because microalgae can be cultured efficiently using light energy, diverse nutrients, and/or carbon dioxide without occupying cultivated farmland or being affected by the season [2]. Notably, these organisms can be grown in different settings, in particular the controlled cultivation for the production of specific high-value chemicals in bioreactors [3]. Therefore, microalgal biomass production and microalgae-based chemical refinery for the preparation of various renewable products, including biofuels, carotenoids, and other high-value chemicals, have gained tremendous attention in recent decades. It can efficiently reduce the burdens of, and facilitate sustainability in, industrial and agricultural systems [4]. It is well-known that the exploitation of diverse high-value products is an efficient strategy to meet the urgent needs of global carbon neutrality and ensure the economic feasibility of the sustainable development of the microalgae industry system [5]. For decades, the research studies have focused on the production of high-value products, including innovation of the germplasm resources, investigation of culture conditions, and the exploitation of downstream high-value products from microalgae [6].

The allenic carotenoid fucoxanthin (FX) is the most abundant marine-derived xanthophyll pigment, widely produced by various brown seaweeds and microalgae [7]. FX is considered to possess wide-ranging health benefits to human beings, comprising anti-inflammatory, antioxidant, anti-obesity, anti-diabetic, and antitumor effects [8]. FX has been incorporated as a functional food component in multiple types of food commodities, including biscuits, by some food manufacturers all over the world [9]. FX is safe for humans or animals [10]. Currently, the demand for FX has dramatically increased in the global market, due to its broad application prospects [11].

Presently, commercial FX is mainly extracted from brown seaweeds, because FX is the major carotenoid in seaweeds with a relatively low cost for its extraction [12].The development of green technology for the extraction of fucoxanthin from diverse brown algae using green solvents has received great attention [13], especially the potential application of natural deep eutectic solvents [14,15]. Compared with brown seaweeds, marine microalgae feature a richer FX content and can be cultured under controlled conditions to produce a specific biochemical product [6,16]. Therefore, marine microalgae are promising alternative feedstocks for the industrial production of FX [5]. To date, fucoxanthin production from microalgal biomass is not commercialized, owing to several technical and cost barriers, including the screening of high-yielding fucoxanthin-producing microalgae, optimum microalgae culture conditions for fucoxanthin accumulation, and the downstream fucoxanthin preparation approach, featuring environmental-friendliness, sustainability, and cost-effectiveness [17]. Among the marine microalgae, the diatom *Phaeodactylum tricornutum*, a well-known model diatom, has a silica frustule surrounded by an organic wall [18], and has been proven to have a strong potential for the production of various high-value products, including eicosapentaenoic acid, FX, and neutral lipids [19]. Notably, *P. tricornutum* is an excellent source of FX owing to (all-*E*)-FX being the predominant carotenoid therein [20]. Tremendous progress has been achieved in developing methods for downstream fucoxanthin preparation from *P. tricornutum* [21,22,23].

As one of the most economically valuable bait microalgae, *Isochrysis zhangjiangensis* is small unicellular phytoplankton [24,25], and is easily cultivated at a large scale indoors and outdoors. It is also a promising microalgal producer of FX, due to its high FX content and cell wall nature. The research studies focus on the extraction, isolation, and purification of FX from the lyophilized *I.* *galbana* [24,26,27]. However, FX extraction and purification from *I. zhangjiangensis* have not been fully investigated.

The exploitation of the separation of FX from *I. zhangjiangensis* using a green chemistry strategy is still facing numerous challenges, which has become the main scientific-technological barrier to the high-value utilization of the microalga. The FX production yield from microalgae depends on the microalgal species, biochemical characterization of algal biomass (including carotenoid and chlorophyll profiles), cell wall nature, and the extraction’s experimental conditions [22]. For example, an efficient method of FX purification from *P. tricornutum* has been developed, using two-step ethanol precipitation under a vacuum evaporation process [23]. However, the high-purity FX cannot be obtained from the microalga *I. zhangjiangensis* according to this method [23], despite multiple attempts. The possible explanation for this result is the microalgal species’ differences. According to the reports, each microalga belonging to a different phyla has its unique compositions of species-specific carotenoid and chlorophyll profiles [20,28]. Additionally, the separation of single compounds remains a challenge, owing to the coexistence of various components with similar polarities in the complex microalgal biomass [29]. Thus, the search for new algal species to meet future fucoxanthin-producing demands is a major challenge; additionally, an effective preparation process for FX should be more specific in targeting the particular algal species and providing an end product FX of a high purity and yield. It is well-known that the downstream preparation for a single value-added product from microalgae is unprofitable, which is a crucial cost-effective step in the actual implementation for future microalgal biorefineries; additionally, the preparation enhances the valorization of the microalgae biomass and provides possible co-optimization operation units for the integrated biorefineries [30]. Therefore, the exploitation of a cost-effective preparation of FX from *I. zhangjiangensis* using a green strategy is a crucial step for the future valorization of microalgae biomass.

Ethanol has been widely used in the preparation of pharmaceutical raw materials. For instance, betalain compounds are successfully extracted from beetroot peel with an aqueous ethanol solvent [31]. As a commonly used purification method, ethanol precipitation has been extensively applied to purify many natural compounds, including proteins and polysaccharides [32,33]. Octadecylsilyl (ODS) column chromatography has been widely used to separate various natural bioactive compounds. For instance, commercially high-purity DHA is successfully refined by industrial HPLC with ODS columns [34]. Considering the economic and environmental aspects, the extraction, separation, and precipitation processes using ethanol as the only organic solvent possess some compelling competitive advantages, such as solvent safety, easy amplification, and eco-friendly products [35]. The two methods also possess the advantage of low production costs, because the majority of ethanol can be reused at industrial levels in multiple methods to reduce the environmental pollution and production costs [36].

In this study, a green and efficient strategy for the production of high-purity FX from *I. zhangjiangensis* was proposed, which possessed several advantages including environmentally friendly technology, green solvent, and high-purity product. It was of great significance for the full use of the *I. zhangjiangensis* microalgal resource and the further exploitation of the related products of FX.

## 2. Results

### 2.1. FX content in the Freeze-Dried I. zhangjiangensis Powder

In the present study, the FX content in the freeze-dried *I. zhangjiangensis* powder was determined by the HPLC analysis. In brief, the methanol extracts of freeze-dried *I. zhangjiangensis* were measured to determine the FX content. The results indicated that the FX content in the freeze-dried *I. zhangjiangensis* was 5.98 ± 0.17 mg/g, which manifested that the microalgae *I. zhangjiangensis* had more FX than the brown seaweeds previously investigated [37]. It is critical to exploit a microalgal species with a high FX content for microalgae-based FX production [38]. Thus, the economically valuable microalgae *I. zhangjiangensis* was a promising potential feedstock for FX production, owing to its high content of FX, rapid growth, and efficient photosynthesis [39,40].

### 2.2. Influence of Different Extraction Conditions on FX Yield 

#### 2.2.1. Influence of Different Solvent Types

**Figure 1:**
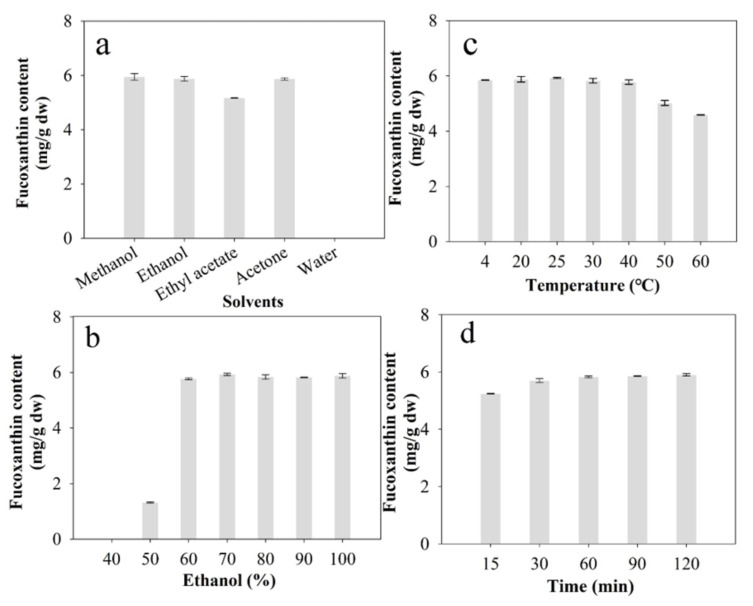
Influence of (**a**) solvent type; (**b**) solvent ratio (ethanol/water, %, *v*/*v*); (**c**) temperature; and (**d**) time on fucoxanthin (FX) extraction from lyophilized *I. zhangjiangensis.* Error bars indicate the standard deviation of three replicates.

Figure 1a shows the effect of five different solvents on the FX yield from *I. zhangjiangensis*. The results indicated that water could not effectively extract FX, which was consistent with the previously published reports dealing with FX extraction from *P. tricornutum* [38]. However, FX could be effectively extracted by three hydrophilic organic solvents, including methanol, ethanol, and acetone, in this study. There was no remarkable difference in the yield of FX among the three hydrophilic organic solvents (*p* > 0.05). A lower amount of FX was found for the ethyl acetate extractions compared with the ethanol-based experiments. This could be attributed to several reasons, the first of which is the polarity of the solvents and the solubility of FX. It is commonly known that the yield of solvent extraction primarily depends on the solubility properties of the targeted molecules with solvents of different polarities that can dissolve the targeted molecules based upon their relative polarity [41]. The polar marine carotenoid FX is easily dissolved in organic solvents with a strong polarity [42]. Second, the efficiency of the FX extraction also depends on the specific cell structural properties of the microalga *I. zhangjiangensis*, which is a small unicellular marine phytoplankton (about 5~7 μm) and possesses structure-based fragility due to the absence of a cell wall [24,43]. FX-chlorophyll protein (FCP) is the core molecular complex and plays a vital role in efficient light-harvesting and light protection [44]. Therefore, *I. zhangjiangensis* does not have a cell wall, which may help explain its easy extraction using hydrophilic organic solvents. In this study, there was no remarkable difference in the extraction amount of FX among the three hydrophilic organic solvents (*p* > 0.05). Overall, ethanol was determined as the best extraction solvent for subsequent optimization studies, owing to its high efficiency, safety, and environmental friendliness [45].

#### 2.2.2. Influence of the Ethanol/Water Mixed Solvent

To evaluate the influence of ethanol to water ratio on fucoxanthin extraction from *I. zhangjiangensis*, different ethanol concentrations (*v*/*v*, 40%, 50%, 60%, 70%, 80%, 90%, and 100%, respectively) were investigated. As presented in Figure 1b, the extraction rate of FX was gradually enhanced with ethanol concentrations between 40% and 70% (*v*/*v*), emphasizing the significance and role of ethanol in FX extraction. However, there was no appreciable difference among 70%, 80%, 90%, and 100% (*v*/*v*) ethanol. Therefore, the results could be attributed to the structural properties of FX and the natural structure-based fragility of the microalga *I. zhangjiangensis* [46]. Generally, an effective extraction for a single product should be more intentional towards the target product, while reducing the co-extracted impurities [17]. According to the report, the lipids yield obtained from the microalgae increases with the content of ethanol [24]. In this study, 70% ethanol is preferable due to its high efficiency of FX and the simultaneous reduction in the co-extracted lipids, which may create favorable conditions for the subsequent FX separation. Therefore, 70% ethanol (*v*/*v*) was selected as the optimal solvent in subsequent experiments.

#### 2.2.3. Influence of Temperature

Temperature is a critical parameter during solvent extraction, because the solubility properties of the targeted molecules and the polarity properties of the solvents may have been changed with increasing temperature [41]. As shown in Figure 1c, there was no remarkable difference in the extraction amount of FX with the temperature in the 4 °C to 40 °C range (*p* > 0.05). This could be mainly attributed to the unique cellular structure of the microalga *I. zhangjiangensis*, which is a unicellular marine phytoplankton species and possesses structural fragility due to the absence of an intact cell wall [24,43]. However, the FX extraction efficiency from the diatom *P. tricornutum* is significantly influenced by the extraction temperature, which could be attributed to the obstacles of the diatom frustules of the *P. tricornutum* [23]. As shown in Figure 1c, the extraction rate of FX gradually decreased with temperatures between 50 °C and 60 °C, reflecting the FX degradation caused by high temperatures in FX extraction [47]. Thus, *I. zhangjiangensis* was a promising feedstock for FX production due to its specific structural features. In this study, 25 °C was determined as the suitable temperature for further optimization, due to its energy saving.

#### 2.2.4. Influence of Extraction Time

Considering the energy and cost consumption of the extraction process, it is crucial to optimize the extraction time. As presented in Figure 1d, as the extraction duration was prolonged, the amounts of FX obtained increased gradually, although the increase was not remarkable after 60 min. The specific cell structural properties of *I. zhangjiangensis* greatly influenced the FX extraction from this species. It is a small unicellular marine phytoplankton (about 5~7 μm) and possesses structure-based vulnerability, owing to the absence of a cell wall [28]. The FX-chlorophyll protein (FCP) is the core molecular complex and plays a crucial role in efficient light-harvesting and protection [44]. Hence, the absence of a cell wall in *I. zhangjiangensis* may facilitate FX extraction within 60 min. To conserve energy and costs, 60 min was selected as the optimum time condition.

#### 2.2.5. Influence of Number of Extractions

**Figure 2:**
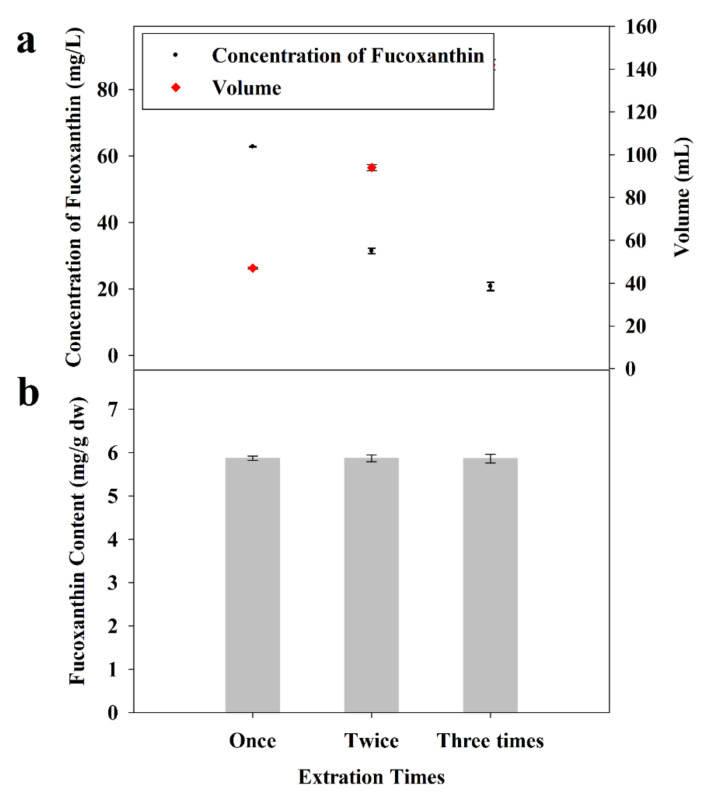
Influence of extraction times on FX extraction from lyophilized *I. zhangjiangensis*. (**a**) The volume of extraction solution and concentration of FX therein; (**b**) Yield of FX. Error bars indicate the standard deviation of three replicates.

The influence of the number of extractions on the extraction efficiency of FX from *I. zhangjiangensis* is shown in Figure 2. In this study, the sharp increase in the volume and the dramatic reduction in the FX concentration in the extraction solution are the results of adding an extraction round. An overwhelming majority (approximately 98%) of the total FX could be effectively extracted by a single extraction. This fact may suggest that the FX could be completely extracted from *I. zhangjiangensis* in one round of extraction. The results were consistent with those of the extraction by a single extraction in ethanol in the report [24]. This could be mainly attributed to *I. zhangjiangensis*, which is a single-celled marine phytoplankton species with a fragile structure owing to the lack of a complete cell wall [24,43]. To conserve the cost of the extraction process, it could be expected that a single extraction was adequate for FX extraction from *I. zhangjiangensis*, due to its high extraction efficiency, time- and solvent-saving. Notably, the extraction solution with a high concentration of FX (62.76 ± 0.51 mg/L) was obtained by a single extraction, which could contribute to the feasibility of the subsequent FX purification. Thus, a single extraction was sufficient.

Therefore, a convenient and low-cost method for extracting FX extraction from lyophilized *I. zhangjiangensis* was developed. The optimized conditions for the FX extraction were as follows: 70% ethanol (*v*/*v*); the solvent-to-material ratio of 100 mL/g; the temperature of 25 °C; extraction time of 60 min; and a single extraction, which resulted in the FX yield of 5.87 ± 0.05 mg/g lyophilized sample, and the recovery efficiency of 98.16 ± 0.84%. The results revealed that *I. zhangjiangensis* was more suitable for fucoxanthin extraction compared with the diatom *P. tricornutum*, due to the higher extraction efficiency. It mainly relied on the specific cell structural properties of *I. zhangjiangensis*, which is a small unicellular marine phytoplankton without a cell wall [24,43]. This is beneficial to several factors related to fucoxanthin extraction, including the ethanol content, extraction temperature, extraction time, and the number of extraction rounds in this study.

### 2.3. Separation of FX by ODS Column Chromatography

**Figure 3:**
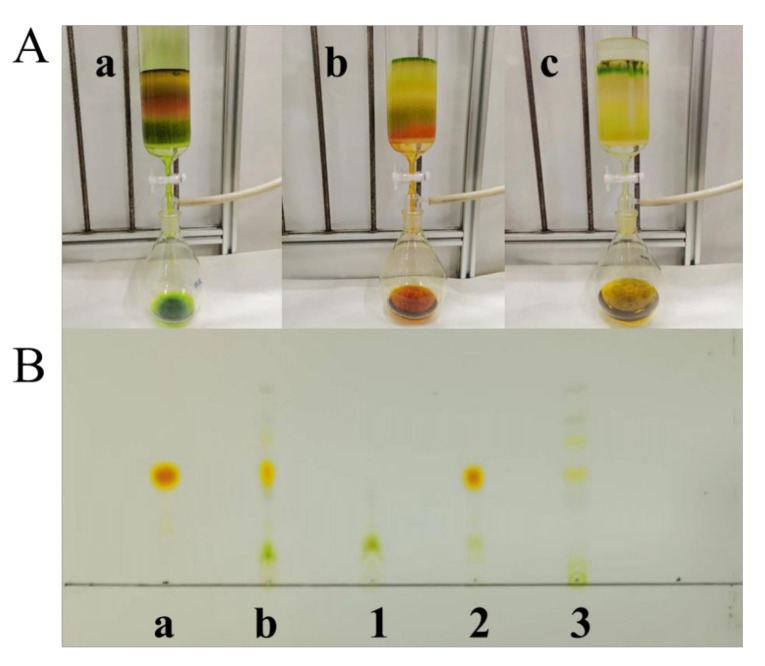
(**A**) Elution process of the extraction solution from *I. zhangjiangensis* with an ODS column chromatography. (**a**) elution using ethanol/water (70:30, *v*/*v*); (**b**) elution using ethanol/water (85:15, *v*/*v*); (**c**) elution using absolute ethanol; (**B**) TLC analysis results: (**a**) FX standard; (**b**) *I. zhangjiangensis* extraction solution; (1) the fraction obtained by ethanol/water (70:30, *v*/*v*); (2) the fraction obtained by ethanol/water (85:15, *v*/*v*); (3) the fraction obtained by absolute ethanol.

Figure 3A indicates the representativeness of the images related to the separation procedure of the FX-containing solution extracted from *I. zhangjiangensis* by an ODS open column chromatography. The representativeness indicated the excellent separation effect of the ODS column chromatography, using a stepwise elution with ethanol/water mixtures (70:30, 85:15, 100:0, *v*/*v*, respectively), which fulfilled the anticipated requirement for the FX separation. Hence, it directly stated that the ODS column chromatography could effectively isolate FX from the FX-containing solution. The excellent separation effect could also be proved by the evidence of the results of HPLC and TLC analysis. The polar impurities were effectively removed (Figure 3B, Sample 1) by the ethanol/water mixture (70:30, *v*/*v*). The results of the HPLC analysis indicated that the recovery efficiency of FX was approximately 0%. Notably, the immense majority of FX was effectively obtained by an ethanol/water mixture (85:15, *v*/*v*), and the recovery efficiency of FX in this FX-rich elute was 89.65 ± 2.85%; additionally, the FX-rich elute contained a high concentration of FX (145.91 ± 1.82 mg/L). The TLC analysis implied that the FX was eluted together with minor impurities (Figure 3B, Sample 2). In this study, the FX-rich fraction gained by the ODS column chromatography may provide a favorable foundation for the subsequent ethanol precipitation, owing to its high FX concentration and minor impurities. The FX separation effect could seriously affect the FX precipitation in the next step. The preparation of minor products from microalgae remains a challenge owing to the coexistence of various components with a similar polarity in the microalgae biomass [29]. The ODS column chromatography has several advantages, the first of which is the ability to reuse the ODS particles loaded onto the ODS open tubular column multiple times. Second, the majority of the ethanol can be recycled at industrial levels in multiple ways. Both of them could effectively reduce the environmental pollution and production costs. Thus, the reduced material (ODS particles) and solvent consumption, along with the excellent separation effect, make this process more feasible on an industrial scale.

### 2.4. Optimization of Ethanol Precipitation

#### 2.4.1. Effect of Ethanol Content on Precipitation

**Figure 4:**
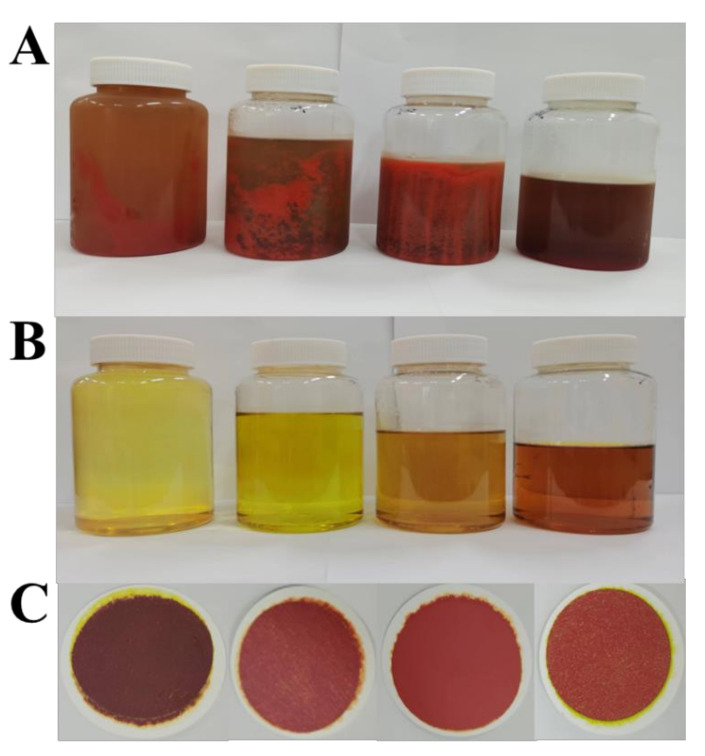
The FX precipitation process from lyophilized *I. zhangjiangensis*. (**A**) FX precipitation after storage at 4 °C for 24 h; (**B**) Supernatants after filtering; (**C**) Precipitates after filtering. Filtration was performed using a 0.45-μm filter membrane. The four samples in groups (**A**–**C**) were obtained from precipitation solutions with different contents of ethanol (from left to right: 30%, 40%, 50%, and 60% ethanol, respectively).

**Figure 5:**
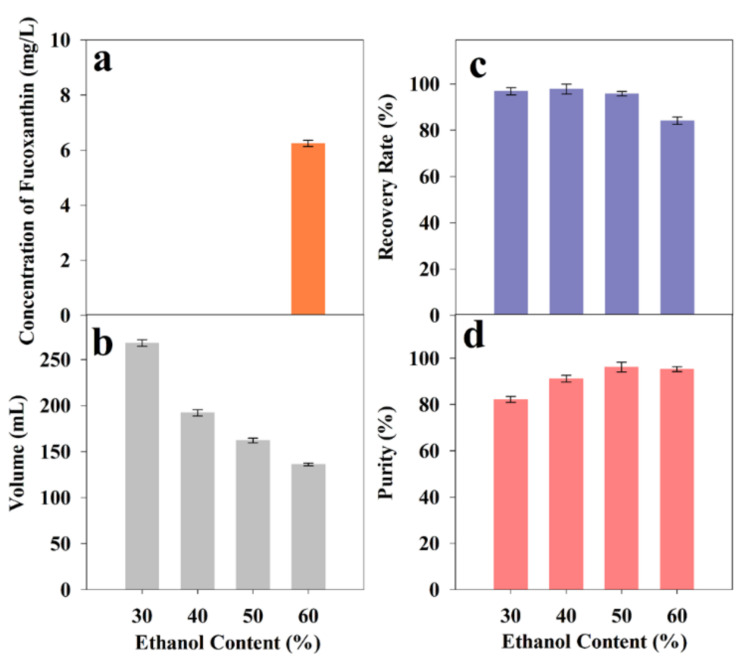
Influence of ethanol content (30%~60%) on FX precipitation after storage at 4 °C for 24 h from lyophilized *I. zhangjiangensis*. (**a**) The concentration of FX in the supernatant; (**b**) The volume of the precipitation solution; (**c**) The recovery rate of FX; (**d**) The purity of FX. Error bars indicate the standard deviation of three replicates.

Refined solid fucoxanthin could be successfully obtained by controlling the ethanol proportion in the solution. Briefly, the FX-rich fraction (concentration of FX 145.91 ± 1.82 mg/L) was added to different amounts of water to adjust the ethanol content in the precipitation system. The images of the FX precipitation and the effects of the ethanol content (30%~60%) on the FX precipitation from freeze-dried *I. zhangjiangensis* are shown in Figure 4 and Figure 5, respectively. The results declared that the FX content in the supernatants with an ethanol content of 30~50% was approximately 0 mg/L, whereas it was 0.85 mg/L in the supernatant with 60% ethanol. The obtained recovery efficiencies of the FX from the solutions with an ethanol content of 30%, 40%, and 50% were more than 95%. The recovery efficiency of the FX obtained from the solution with 60% ethanol was 84.08 ± 1.62%. Based on the report, the ethanol content of the ethanol precipitation supernatant influences the solubility of the components inside [35]. In this study, the solution containing 60% ethanol was unfavorable, as it caused the FX loss due to a small amount of the FX dissolved in the supernatant.

The results indicated that the purity of the FX was enhanced with the ethanol content in the range of 30% and 60%. Notably, the purity of the FX precipitated from the two solutions with an ethanol content of 50% and 60% was more than 95%. In general, the lipids’ solubility is increased with the ethanol content [36]. In this study, properly enhancing the ethanol content is favorable, as it will facilitate the removal of fat-soluble impurities. Therefore, 50% ethanol was determined as the optimum condition for further optimization studies, owing to the high purity and high recovery efficiency of the FX.

#### 2.4.2. Effect of Temperature on Precipitation

**Figure 6:**
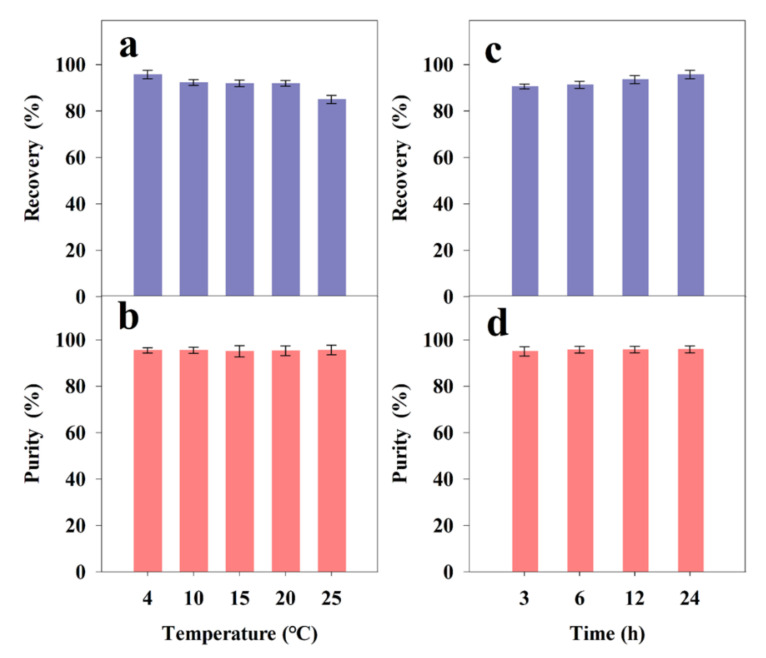
Influence of temperature (4~25 °C) and precipitation time (3~24 h) on FX precipitation from lyophilized *I. zhangjiangensis*. (**a**) The recovery rate of FX at 4~25 °C; (**b**) The purity of FX at 4~25 °C; (**c**) The recovery rate of FX in 3~24 h; (**d**) The purity of FX in 3~24 h. Error bars indicate the standard deviation of three replicates.

The effects of the temperatures (4~25 °C) on the FX precipitation after storage for 24 h from freeze-dried *I. zhangjiangensis* are shown in Figure 6a,b. The results implied that the FX recovery efficiency was gradually increased with the decrease in the precipitation temperature. It is well known that FX is an unstable compound, and is easily degraded when exposed to air, heat, and light [43]. In this study, the results also confirmed that the precipitation temperature was a critical parameter because it affected the solubility and stability of the FX [35]. Interestingly, the purity of the FX obtained under the five temperatures was more than 95%. In this study, 4 °C was selected as the precipitation condition for subsequent optimization experiments because of its high recovery of FX.

#### 2.4.3. Effect of Precipitation Time on Precipitation

The precipitation effects of the times (3~24 h) on FX precipitation from freeze-dried *I. zhangjiangensis* are shown in Figure 6c,d. The results indicated that the FX recovery efficiencies were gradually increased with the extension of the precipitation time. According to a report, the precipitation time is considered a key parameter and has an important effect on the effect of the ethanol precipitation process [35]. The results implied that storage for 24 h contributed to the entire precipitation of the FX (recovery efficiency of FX 95.77 ± 1.81%). Moreover, the purity of the FX obtained from 3~24 h of storage was more than 95%. Therefore, 24 h was recommended, due to its high recovery efficiency and purity of FX.

In the experiment, an efficient method for FX purification from freeze-dried *I. zhangjiangensis* was established, using ethanol precipitation, and the optimized precipitation conditions were as follows: 50% ethanol; precipitation temperature of 4 °C; and precipitation time of 24 h. Under these optimized conditions, the purity of FX was 95.91 ± 1.51%, and the recovery efficiency was 95.77 ± 1.81%.

In conclusion, under the optimized extraction, ODS column chromatography, and ethanol precipitation conditions, the total recovery efficiency of FX was 84.28 ± 2.56%, and the purity of FX was 95.91 ± 1.51%. As a commonly used purification method, the ethanol precipitation has been extensively applied to purify many natural compounds, including proteins and polysaccharides [32,33]. The ethanol precipitation of FX had the potential for large-scale industrialization, due to its solvent safety, ease of scale-up, and sustainable products; additionally, most of the ethanol used in the study could be reused. Considering the reduction in the environmental pollution and production costs, the advantages of ethanol precipitation in this study make it attractive and useful.

### 2.5. Identification of FX

HPLC was used to analyze FX from *I. zhangjiangensis* (Figure S1, Supplementary Materials). The purified product was determined as FX according to the fragment pattern at m/z 659.4, which corresponded to [M + H]^+^ (Figure S2, Supplementary Materials). The purified product was subjected to 1D NMR spectroscopy and determined as all-trans FX, based on the literature (Figure S3, Supplementary Materials) [48].

Marine microalgae are sustainable multipurpose feedstocks for biofuels and FX owing to their high biomass productivity and abundant source of FX. Each microalga belonging to different phyla has its unique compositions of species-specific carotenoid and chlorophyll profiles. The FX production from microalgae depends on the biochemical components (including carotenoid and chlorophyll profiles) and other factors, such as extraction conditions, cell wall nature, and FX content in the species of marine microalgae. For example, an efficient method of FX purification from *P. tricornutum* has been developed using a two-step ethanol precipitation under a vacuum evaporation process. However, the high-purity FX could not be obtained from *I. zhangjiangensis* according to this method, despite multiple attempts. It was observed that the FX extracted from *I. zhangjiangensis* was dramatically degraded during the vacuum evaporation process at 25 °C. The result could be attributed to two reasons. First, the flash boiling phenomenon was observed when the extraction solution of *I. zhangjiangensis* was concentrated under vacuum evaporation at 25 °C, which could extend the concentration time, resulting in FX degradation. The differences in the metabolic pathways between the two microalgae *P. tricornutum* and *I. zhangjiangensis* led to significant differences in the secondary metabolites, including pigment composition and the content in the two microalgae, which had an impact on the FX extraction methods, isolation, and purification processes. The impurities co-extracted with FX were different between the two microalgae, *P. tricornutum* and *I. zhangjiangensis*, which influenced the FX purification process during the vacuum evaporation process at 25 °C. Moreover, there are few reports on FX extraction from *I. zhangjiangensis*. In the study, an innovative approach for FX preparation from *I. zhangjiangensis* was established for the first time, which consisted of four steps, including the culture of microalga, ethanol extraction, ODS column chromatography, and ethanol precipitation. The isolation and purification of a single compound from the microalgal biomass is the most expensive step, and is an outstanding question for the actual implementation of microalgal biorefineries. Considering the reduction in the pollution and production costs at a commercial level, the integrated processes, including the culture of microalga, extraction, isolation, and purification should possess several features, including sustainably and cost-efficiency. In this study, *I. zhangjiangensis* was proved as a promising feedstock for FX production, owing to its structural properties of the absence of intact cell walls and high FX content. The methods of extraction, isolation, and purification of FX from *I. zhangjiangensis* are based on a green and cost-efficiency strategy; additionally, the method used ethanol as the only organic solvent, with a high recovery efficiency, and the end product of FX had a high purity, making the overall process commercial on an industrial scale. Therefore, due to the great potential of the high-purity FX in the food and pharmaceutical industry, the current results might facilitate the development of this production method on a commercial scale and enhance the full use of *I. zhangjiangensis.*

## 3. Materials and Methods

### 3.1. Strains and Cultural Conditions

The *I. zhangjiangensis* (NMBjih021-6) was obtained from the Microalgae Collection at Ningbo University. The algal cells were cultured in 100-L plastic cylinders (0.55 m × 0.5 m, height × diameter) at 25 °C, the water body was 80 L, and was continuously aerated at 5 L/min by an oxygen supply pump. The illumination was supplied from above the cylinders at an intensity of about 54 μ mole photons•m^−2^•s^−1^ by 60-W fluorescent lamps. The algal cells were cultured in an f/2 medium composed of filtered and sterilized seawater. After cultivation for 10 days, all of the cultures were collected to centrifuge at 9000 rpm for 30 min at 4 °C. Then, the sedimented cells obtained were washed with deionized water two times. The microalgae cells were lyophilized and preserved at −20 °C for further FX extraction and analysis.

### 3.2. Extraction and Quantitative Analysis of FX from I. zhangjiangensis

The quantitative analysis of the FX was performed based on a previously published method, with a few modifications [49]. Briefly, a quantity of 20 mg of freeze-dried microalgae powder was extracted, using a magnetic stirrer for 2 h with 8 mL absolute methanol in an ice bath under dimmed light. The FX-containing supernatant was gathered by centrifugation at 8000 rpm (30 min, at 4 °C) and sequentially filtered with a 0.22-μm filter membrane. Then, the filtrate was appropriately diluted with absolute methanol and determined by HPLC. To avoid the degradation of the FX, all of the extraction processes were performed in darkness.

The FX content was determined, as previously published with a few modifications [49]. Briefly, a 1260 Infinity II high-performance liquid chromatography system (HPLC) was adopted. The separation of the FX was performed by a YMC C-30 carotenoid column (250 × 4.6 mm ID, 3-μm particle size; Waters, Ireland). The mobile phase consisted of methanol (A) and water (B). The gradient elution program for FX was described as follows: 0–20 min, 90–100% A; 20–25 min, 100% A. The flow velocity was 0.7 mL/min, and the temperature of the column was 35 °C. The injection volume of a sample was 10 μL and detected at 450 nm. The FX standard (purity ≥ 95%, HPLC) was purchased from Sigma-Aldrich Co. Ltd (Sigma-Aldrich, Shanghai, China). The fucoxanthin standard calibrate curve is shown in Figure S4 (Supplementary Materials).

### 3.3. Optimization of FX Extraction from I. zhangjiangensis

In this study, the optimized FX extraction conditions from freeze-dried *I. zhangjiangensis* were established using the one-factor-at-a-time (OFAT) approach [50]. Around 0.5 g of lyophilized *I. zhangjiangensis* was extracted once with 50 mL solvent in a centrifugation tube on a shaking incubator, and was shaken at 150 rpm in darkness for 120 min at 25 °C. Subsequently, the extracted solution was filtered using an organic membrane filter (0.22-μm), and then injected into the HPLC system for FX analysis. The condition parameters affecting extraction were optimized, including the different solvents of different polarity (methanol, ethanol, acetone, ethyl acetate, and water, respectively), the ethanol/water mixed solvents (ethanol concentration, 60%, 70%, 80%, 90%, and 100%, respectively), temperature (4 °C, 20 °C, 25 °C, 30 °C, and 40 °C, respectively), extraction duration (15 min, 30 min, 60 min, 90 min, and 120 min, respectively), and the number of extractions (once, twice, and three times, respectively). The obtained FX was identified and quantified by HPLC analysis. The samples were placed away from light during the whole extraction procedure. All of the experiments were performed independently in triplicate.

### 3.4. ODS Column Chromatographic Experiments

The lyophilized *I. zhangjiangensis* (6 g) was extracted under the optimum extraction conditions in the study. The optimized conditions for FX extraction were as follows: 70% ethanol (*v*/*v*); the solvent-to-material ratio of 100 mL/g; the temperature of 25 °C; extraction time of 60 min; and a single extraction. After the extraction procedure, the mixture was high-speed centrifuged at 8000 rpm for 30 min at a low temperature of 4 °C to remove the depleted biomass. The supernatant obtained by centrifugation was collected for subsequent FX isolation, using an ODS open column chromatography protocol combining gradient elution. In brief, the FX-containing extraction solution (approximately 580 mL) was added to an ODS open tubular column (30 cm × 7.5 cm i.d.) loaded with ODS particles (250 g dry weight, YMC^®^ GEL ODS-A-HG S-50 μm). The sample was eluted into three fractions at a flow velocity of 1.5 mL/s, using a stepwise elution with ethanol/water mixtures (70:30, 85:15, 100:0, *v*/*v*, respectively). The eluate obtained by the 70% (*v*/*v*) ethanol/water mixture was approximately 1300 mL. Then, the FX-rich fraction (around 220 mL) eluted by 85% (*v*/*v*) ethanol/water mixture was collected for the subsequent FX precipitation. The rest was eluted by the absolute ethanol. The eluate of each part was collected and analyzed by HPLC and thin-layer chromatography (TLC). The amounts of FX for each eluted fraction were assessed, and then the FX efficiencies were calculated.

### 3.5. Optimization of Ethanol Precipitation

The FX was purified by ethanol precipitation. Briefly, a 100 mL FX-rich fraction containing 145 mg/L FX was added with water to set ethanol content and stored at the set temperature. The precipitation of the FX was gradually formed with the extension of the standing time. At the end of precipitation, the precipitates were recovered by filtration with a 0.45-μm membrane filter and washed with deionized water at a solid to solvent ratio of 1:10 (w/v) three times. The refined solid FX was freeze-dried and then sealed. The condition parameters affecting FX precipitation were optimized, including ethanol content (30%, 40%, 50%, and 60%, *v*/*v*, respectively), precipitation temperature (4 °C, 10 °C, 15 °C, 20 °C, and 25 °C, respectively), and precipitation time (3 h, 6 h, 12 h, and 24 h, respectively).

### 3.6. TLC Analysis of FX

The eluates obtained by ODS column chromatography were analyzed by TLC. The TLC analyses were performed with analytical-grade organic reagents and high silica gel prefabricated GF254 plates. A capillary syringe was used in the study to load the sample onto the TLC. After drying the samples, the TLC was developed in a glass chamber which contained the chromatographic developing solvent with acetone to petroleum ether (boiling range 60~90 °C) ratio of 1:2 (*v*/*v*). As soon as the samples were isolated on the plates and the developing reagent reached its expected position, the TLC plates were removed from the developing chamber and photographed immediately under natural light.

### 3.7. Identification of FX from I. zhangjiangensis

The electrospray ionization mass spectrometry (ESI-MS) was carried out by an electrospray ionization-quadrupole-time of flight mass spectrometry (ESI-Q-TOF MS; Waters, Milford, CT, USA). The Proton Magnetic Resonance (^1^H-NMR) spectra were created by an AVANCE 600 MHz NMR spectrometer at a 298 K field, in the presence of tetramethylsilane as the internal standard (Bruker, Fällanden, Switzerland).

### 3.8. Statistical Analysis

All of the experiments were conducted in triplicate, and the error bars indicate the standard deviation. All of the data were analyzed by the IBM SPSS 26 package. A *p* < 0.05 was considered statistically significant. The statistical comparisons were analyzed by the least significant difference (LSD) test.

## 4. Conclusions

The current study establishes a green and efficient strategy for the production of high-purity FX from *I. zhangjiangensis* and highlights the microalga as a promising feedstock for FX production. The ODS-based column chromatography with the solvent elution system of ethanol/water is efficient due to the collection of the FX-rich elute containing a high concentration of FX and minor impurities. The precipitation of FX based on ethanol/water mixtures is efficient and achieves a high purity. What is more, the method proposed in the current study could easily be amplified at a commercial level.

## Supplementary Materials

The following are available online at https://www.mdpi.com/article/10.3390/md20080510/s1, Figure S1: Typical chromatograms of samples obtained in this study; Figure S2: The positive ESIMS spectrum of fucoxanthin derived from the solution with 50% ethanol after storage at 4 °C for 24 h; Figure S3: ^1^H NMR (600 MHz, CDCl_3_) spectrum derived from the solution with 50% ethanol after storage at 4 °C for 24 h; Figure S4: Standard curve of fucoxanthin.
