# Supplementary material for: High-Purity Fucoxanthin Can Be Efficiently Prepared from Isochrysis zhangjiangensis by Ethanol-Based Green Method Coupled with Octadecylsilyl (ODS) Column Chromatography
Source: Mar Drugs. 2022 Aug 11;20(8):510. doi: 10.3390/md20080510 (PMC9410198; doi:10.3390/md20080510)
Supplement: Supplementary file 1 [file marinedrugs-20-00510-s001.zip › marinedrugs-1836931-supplementary.pdf]

Supplementary material

High-purity fucoxanthin can be efficiently prepared from *Isochrysis zhangjiangensis* by ethanol-based green method coupled with Octadecylsilyl (ODS) column chromatography

GengJie Zhuang[a,1], Yuemei Ye[a,1], Junling Zhao[a], Chengxu Zhou[a], Junwang Zhu[a], Yanrong Li[b], Jinrong Zhang[a,*], Xiaojun Yan[c,*]

[a] *College of Food and Pharmaceutical Sciences, Ningbo University, Ningbo 315211, China*

[b] *Ningbo Institute of Oceanography, Ningbo, Zhejiang 315832, China*

[c] *Key Laboratory of Applied Marine Biotechnology of Ministry of Education, Ningbo University, Ningbo 315211, China*

[*]Corresponding author; E-mail address: zhangjinrong@nbu.edu.cn (J. Z.); yanxiaojun@nbu.edu.cn (X. Y.)

[1] These authors contribute equally to this study.

(a)
(b)
(c)
(d)

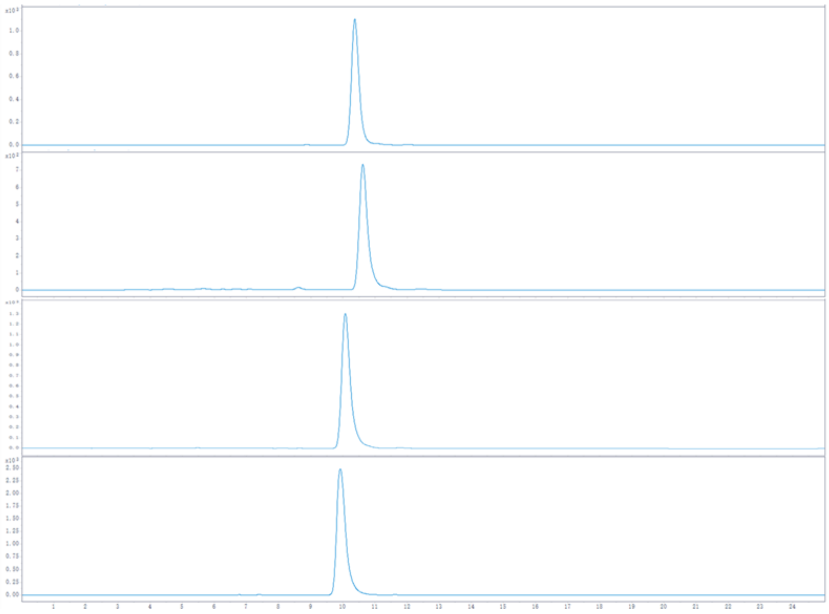

Figure S1 Typical chromatograms of samples obtained in this study. (a) the standard fucoxanthin with a concentration of 100 mg/L; (b) the *I. zhangjiangensis* extract solution; (c) the FX-rich fraction obtained by ethanol/ water (85: 15, v/v); (d) the purified fucoxanthin with a concentration of 308 mg/L.

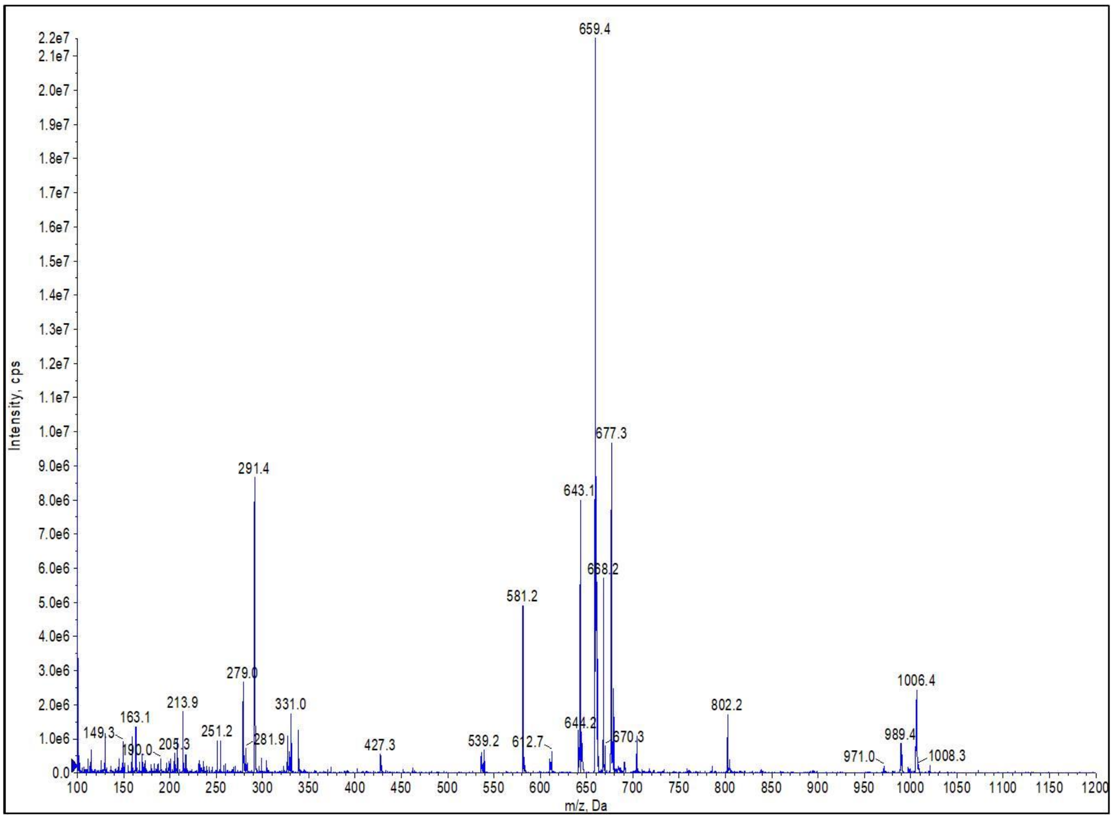

Figure S2 The positive ESIMS spectrum of fucoxanthin derived from the solution with 50% ethanol after storage at 4 ◦C for 24 h.

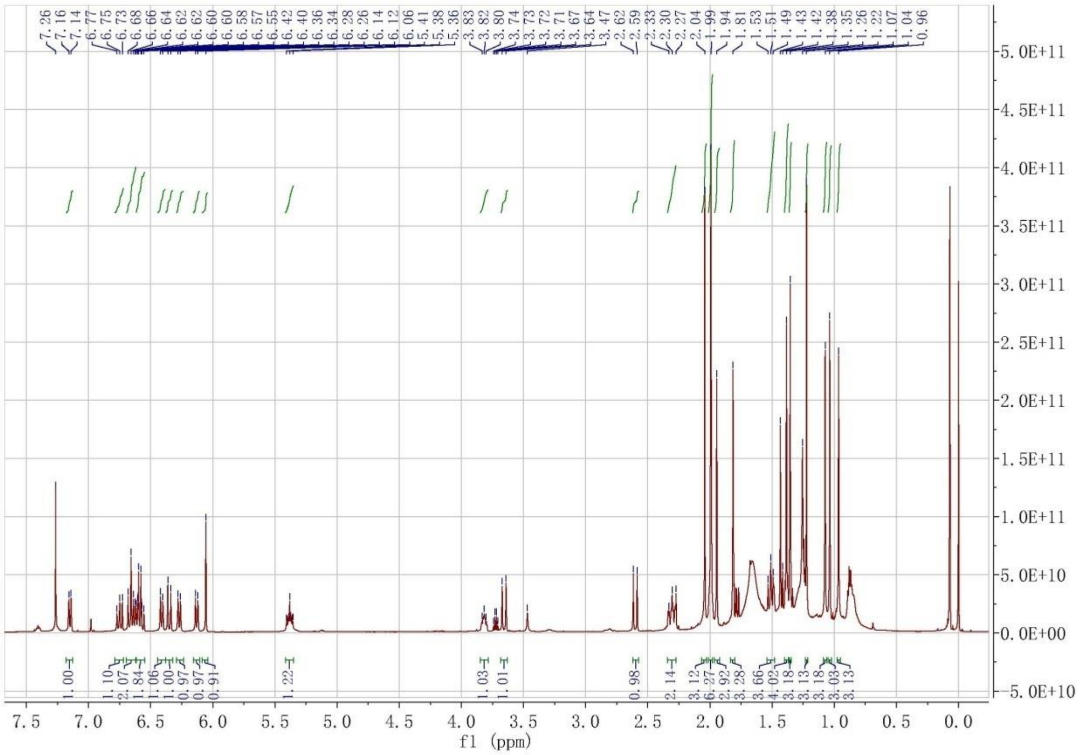

Figure S3 ^{1}H NMR (600 MHz, $CDCl_3$) spectrum of fucoxanthin derived from the solution with 50% ethanol after storage at 4 ℃ for 24 h.

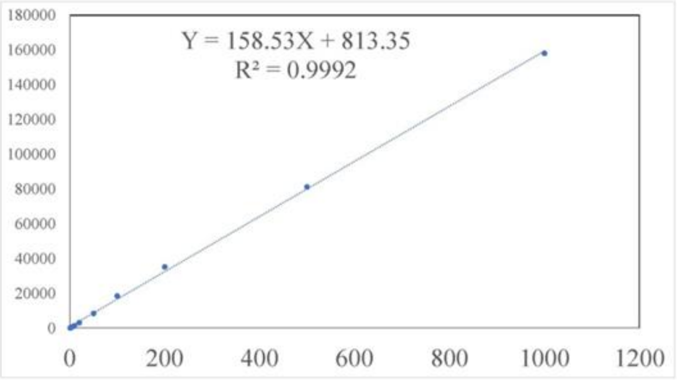

Figure S4 Standard curve of fucoxanthin

The prepared standard solutions were used to construct the calibration curve, and the linearity of the curve was determined by the corresponding correlation coefficient. A calibration curve of fucoxanthin was built within 1-1,000 μg/mL. The limit of quantitation was 0.01 μg/mL. The mean linear regression equation was $Y=158.53X+813.35$ with a correlation coefficient of 0.9992, where Y is the peak HPLC area for fucoxanthin, and X is the concentration of fucoxanthin (μg/mL).
